# Intranasal monkeypox marmoset model: Prophylactic antibody treatment provides benefit against severe monkeypox virus disease

**DOI:** 10.1371/journal.pntd.0006581

**Published:** 2018-06-21

**Authors:** Eric M. Mucker, Suzanne E. Wollen-Roberts, Adrienne Kimmel, Josh Shamblin, Darryl Sampey, Jay W. Hooper

**Affiliations:** 1 United States Army Medical Research Institute of Infectious Diseases, Virology Division, Fort Detrick Maryland, United States of America; 2 BioFactura, Inc, Frederick, Maryland, United States of America; University of Texas Medical Branch, UNITED STATES

## Abstract

Concerns regarding outbreaks of human monkeypox or the potential reintroduction of smallpox into an immunological naïve population have prompted the development of animal models and countermeasures. Here we present a marmoset model of monkeypox and smallpox disease utilizing a relevant poxvirus via a natural exposure route. We found that 1000 plaque forming units (PFU) of Monkeypox virus was sufficient to recapitulate smallpox disease, to include an incubation period of approximately 13 days, followed by the onset of rash, and death between 15 and 17 days. Temporally accurate manifestation of viremia and oral shedding were also features. The number of lesions ranged from no lesions to 299, the most reported in a marmoset exposed to a poxvirus. To both evaluate the efficacy of our antibodies and the applicability of the model system, marmosets were prophylactically treated with two monoclonal antibodies, c7D11 and c8A. Of three marmosets, two were completely free of disease and a single marmoset died 8 days after the mock (n = 1) or PBS control(s) (n = 2). Evaluation of the serum levels of the three animals provided a possible explanation to the animal succumbing to disease. Interestingly, more females had lesions (and a greater number of lesions) and lower viral burden (viremia and oral shedding) than males in our studies, suggesting a possible gender effect.

## Author summary

Poxviruses are large viruses capable of causing disease in humans. Of the known poxviruses, variola virus and monkeypox virus cause the most severe disease, smallpox and human monkeypox, respectively. Models utilizing these viruses are valuable for the study of host-virus interaction and for evaluating countermeasures. Arguably the best models utilizing variola virus or monkeypox virus involve the intravenous inoculation of nonhuman primates. Because these models have an unnatural route of infection and bypass the incubation of the disease, more genuine models are sought. Here we tested whether marmosets could be infected with monkeypox virus via a natural route and, if so, whether the model could be applied to study countermeasures. We found that the animals were susceptible to monekypox virus at a low dose via a natural route, uncannily mirrored human disease onset, and could be protected by prophylactic treatment of two novel monoclonal antibodies. Furthermore, we found potential disease disparity between males and females that, if proven true, could stimulate the exploration of a mechanistic explanation to describe the disparity and deepen our understanding of poxvirus pathogenesis, immunology and/or marmoset biology.

## Introduction

Poxviruses are large and complex viruses and belong to the *Family Poxviridae*. All share a double stranded DNA genome and replicate in the cytoplasm. Members of the genus Orthopoxvirus are arguably the most recognizable poxviruses as they include variola virus, monkeypox virus, and vaccinia virus, the causative agent of smallpox, monkeypox, and the virus converted to a vaccine to eradicate smallpox from nature, respectively. Because of the lack of, or waning, immunity due to the cessation of vaccination, there are concerns about our vulnerability to natural (zoonotic) or man-made poxviral threats.

For the most part, human monkeypox is an endemic disease in Africa. In 2003, multiple cases of monkeypox were reported within the U.S., representing the first confirmed incidence of monkeypox breaching the African continent [1]. Within Africa, there continue to be outbreaks that are seemingly more intense. Reports show an estimated 20 fold increase in monkeypox incidence relative to the vaccination era [2–4]. Data collected from the Bokungu Health Zone suggest that large changes (as much as 6 fold) can occur within a two year period [3]. Currently, outbreaks in Nigeria and Republic of Congo have been upgraded (as an emergency) by risk assessments performed by the World Health Organization’s Emergency Response Framework.

Unlike monkeypox virus, variola virus is host restricted to humans and is not zoonotic. Because of this, the eradication of smallpox disease was possible through intense survellience and vaccination efforts. Variola virus, was not eradicated and still resides in at least two locations in the world, the Centers for Disease Control and Prevention, Atlanta, Gerorgia, United States and State Research Center of Virology and Biotechnology, Novosibirsk, Russia. Whether accidental, such as the potential harm from the 6 unaccounted vials of variola virus found at a U.S. Food and Drug Administration facility[5], or through the auspices of a rogue government or organization, the release of variola virus on a suceptable population would be catastrophic. Furthermore, while we still have the threat of a radical state or individual obtaining Variola virus stocks, the concern has shifted to the capability of the mal-intent to synthesize viral stocks [6].

Both monkeypox and smallpox have a similar disease presentation in humans. After an approximate one to two week incubation period, both diseases present with general symptoms (*i*.*e*., fever, headache, malaise). Unlike smallpox, lymphadenopathy is commonly reported for monkeypox during this prodromal phase. A progressive rash ensues. Again, the extent and severity of the rash tends to be attenuated in monkeypox cases. Overall, monkeypox is likely to have a more favorable outcome, but, the extent and presentation of disease will likely depend on many factors (*e*.*g*.,strain, route of exposure, host immune status)

Due to the threats imposed by monkeypox and smallpox, medical countermeasures are being developed. Currently there is only one U.S. Food and Drug Administration (FDA) licensed product with an indication for smallpox disease, the ACAM2000 vaccine. The benefit-to-risk ratio with ACAM2000 is very small and was a major factor for cessation of vaccinating after the eradication of smallpox disease. In fact, vaccinia immune globulin (VIG) was licensed to counteract the adverse events associated with the smallpox vaccine. Being that a significant portion of the United States is contraindicated for our only line of defense against monkeypox or smallpox, safer vaccines [7–9] and therapeutics [10–12] are being developed.

Here, we describe a model for the study of human monkeypox (and smallpox) and present the first application of any marmoset (*Callithrix jacchus*) model for the treatment of poxvirus disease. More specifically, we used a cocktail of two human-chimeric monoclonal antibodies, c7D11 [13] and c8A [14] and evaluated the ability of the antibodies to prophylactically protect against a lethal dose of monkeypox virus. The antibodies, 7D11 and c8A, target two morphologically distinct forms of the virus known as the mature virion (MV) and the extracellular virion (EV), respectfully. Inhibiting both forms of the virus is thought necessary for optimal protection [15–18] This is the first description of a nonhuman primate (NHP) model that utilizes a low dose and natural route of monkeypox virus while still recapitulating the temporal onset of clinical and virological events as described for smallpox. Furthermore, this is the first application of the model (or similar marmoset models) for evaluation of poxviruses countermeasures.

## Methods

### Viruses and cells

Monkeypox virus strain Zaire (V79-I-005) was propagated and titrated as previously described [19, 20]. The virus was the same lot as utilized by Mucker, *et al*. [21]. BS-C-1 cells (ATCC CL-26) were utilized for virus titration of blood and swabs. Inoculums were prepared in phosphate buffered saline (PBS, Gibco)(pH 7.4).

### Monoclonal antibodies

The discovery and characterization of the antibodies are reported elsewhere [13, 14]. For our studies, human-murine (c7D11) and human-chimpanzee (c8A) chimeric antibodies were designed and produced by BioFactura (Frederick, MD). Briefly, both antibodies sequences were constructed utilizing murine light and heavy chain signal peptides and human IgG1 heavy constant sequences. In terms of light chain constant sequences, c7D11 was comprised of human Kappa and c8A human Lambda domains. Stable NS0 cell lines were developed at BioFactura, Inc. using the *StableFast* platform and the single cholesterol selection strategy as described by Sampey, *et*. *al*. [22]. Briefly, bicistronic expression plasmids were constructed coding for both heavy and light chains of either the c7D11 or c8A mAb. Each chain coding sequence was flanked upstream by cytomegalovirus (CMV)-derived promoters and downstream by bovine growth hormone (BGH) polyadenylation sequences (poly-A) comprising independent heavy and light chain expression cassettes. The cholesterol selection marker 17β-hydroxysteroid dehydrogenase type 7 or Hsd17β7 was regulated by an SV40 promoter and SV40 poly-A. The serum-free medium adapted cholesterol auxotrophic NS0 host cell line (NS0-SF, ECACC, Cat No. 03061601) was transfected by electroporation with either the c7D11 or c8A expression plasmids and stable cell lines were selected by withdrawal of exogenous cholesterol. Best performing clones for each mAb were scaled to 3.5L (BioBLU 5c) or 10L (BioBLU 14c) working volume Eppendorf/New Brunswick Scientific CelliGen BioBLU single-use bioreactor systems (Hamburg, GE). Bioreactors were operated in fed-batch mode for 7–9 days and were subsequently harvested and purified by a single Protein A capture step. Final purified mAbs were dialyzed into 1x PBS and sterile filtered.

Antibodies were combined 1:1 prior to subcutaneous injection into NHPs. Isotype control antibody contained identical constant human heavy and light chains as the c7D11 mAb, but with different murine heavy and light chain variable domains that bind an unrelated filovirus glycoprotein.

### Virus titration

Samples (plasma, EDTA whole blood, and throat swabs) were subjected to three freeze-thaw and sonication cycles and plaque titrated as reported by Golden, *et al*. [7]. For assays involving blood samples, dilutions were made in PBS lacking magnesium and calcium (Gibco) and cells washed (pre and post adsorption) with the same PBS.

### Virus neutralization assays

Virus from animal #9, D25 oral swab was used to perform two types of assays. Briefly, virus was diluted and incubated with a constant concentration of c7D11 and adsorbed. Samples incubated with c7D11 were incubated for 1h at 37°C, adsorbed for 1h, and overlayed with Minimal Eagle Medium (MEM, Corning) containing 5% heat inactivated fetal bovine serum, FBS (Gibco).

### Blood processing and hematology

Whole blood samples were collected in Sarstedt K3E Micro Tubes and analyzed by either the AcT Diff 3 (Coulter) or a Hemavet HV950 (Drew Scientific). Plasma was collected by centrifugation at 1800xg for 10 minutes.

### Nonhuman primate treatment and inoculation

Both male and female adult marmosets (*Callithrix jacchus*) weighing between 233–437 grams, were exposed via the intranasal route by instilling (pipet) 50μL of virus into each nare (100μL total) while animal was inhaling. Physicals, lesion counts, weights, rectal temperatures, oral swabs, and venous blood collections were performed previous to, and every three days following, exposure. Animals were anesthetized with isoflurane for all animal manipulations, and prior to euthanasia were anesthetized with Telazol before administration of an intravenous barbiturate overdose.

For the passive transfer experiment, 20mg/kg of each antibody was administered via the subcutaneous route (one injection) 24 hours prior to exposure. Two control animals received an equivalent volume of PBS, while a third received 40mg/kg of a human chimeric, non-poxvirus targeted monoclonal antibody (BioFactura). Survival curve comparsions (Log-rank (Mantel-Cox) test) were performed usng Graphpad Prism.

### Quantitative ELISA

Plates were coated with poxvirus antigen B5, L1 or diluent (bicarbonate buffer, pH 9.6) and incubated at 4°C until assay was performed (1–3 days). Recombinant 6xHis-tagged soluble poxvirus antigens B5 and L1 were transiently expressed in HEK-293T cells and purified by cobalt affinity chromatography (HisPur Cobalt Purification Kit, ThermoFisher, NY). Antigen B5 is the product of the vaccinia virus gene B5R and is the target of monoclonal antibody c8A and antigen L1 is the product of the vaccinia virus gene L1R and is the target of monoclonal antibody c7D11. On the day of the assay, wells of the plate were aspirated and washed with PBS with 0.05% sodium dodecyl sulfate (SDS) and subsequently blocked for two hours with blocking buffer (PBS with 5% skim milk and 0.05%SDS). Wells were washed with PBS with SDS before samples were added. Samples were first diluted into 5% skim milk with SDS and serially diluted 1:5. The standard antibody was diluted 1:10 into marmoset sera (Bioreclamation) and serially diluted 1:5, leaving a single tube that contained only marmoset serum. The spiked and control marmoset sera were diluted in blocking buffer analogous to the samples. Samples were added to the plate in triplicate (two on wells containing antigen and one with no antigen). Plates were sealed and incubated at 37°C for one hour before being mechanically washed. Horseradish peroxidase labeled, anti-human IgG detection antibody (KPL,) diluted in blocking buffer at 1:8000 was then added to each well and incubated at 37°C. After washing,3,3’,5,5’- tetramethylbenzidine (TMB) Sureblue Reserve (KPL) was added, followed by TMB Stop solution (KPL). Absorption readings were performed on a Tecan plate reader (450nm). The data was normalized and values interpolated using curve fit (Sigmoidal, 4PL) on Graphpad Prism. Antigens and monoclonal antibodies c8A and c7D11 were provided by Biofactura.

### Ethics statement

Research was conducted in compliance with the Animal Welfare Act, PHS Policy, and other Federal statutes and regulations relating to animals and experiments involving animals. The facility where this research was conducted was accredited by the Association for Assessment and Accreditation of Laboratory Animal Care, International and adheres to principles stated in the Guide for the Care and Use of Laboratory Animals, National Research Council, 2011. All animal experiments were approved by USAMRIID’s Institutional Animal Care and Use Committee.

Animals were housed in individual metal cages meeting current standards for the duration of the housing period in animal biocontainment level 3. Room environment was centrally controlled by an HVAC system that maintained room humidity and temperature. Animals were provided pelleted commercially available feed and potable water was provided at libitum from an automatic watering system. In addition, animals received supplemental foods, treats and fruits daily. Animals were provided manipulanda (toys, metal mirrors), foraging devices, treats and fruits as enrichment. Treats and extra fruits were increased while in biocontainment. Euthanasia was performed when the animal(s) met the criteria for euthanasia using a score sheet for intervention or when found moribund [21]. The scoring system was based on recumbency, prostration, dyspnea, and responsiveness. Animals were observed and scored at least twice daily by trained study personnel. In addition, husbandry and general assessments were conducted at least once daily by Veterinary Division personnel. Animals requiring euthanasia were anesthetized and subsequently euthanized with a pentobarbital based solution following the AVMA Guidelines on Euthanasia.

## Results

### Dose selection of intranasal monkeypox virus in marmosets

**Fig 1:**
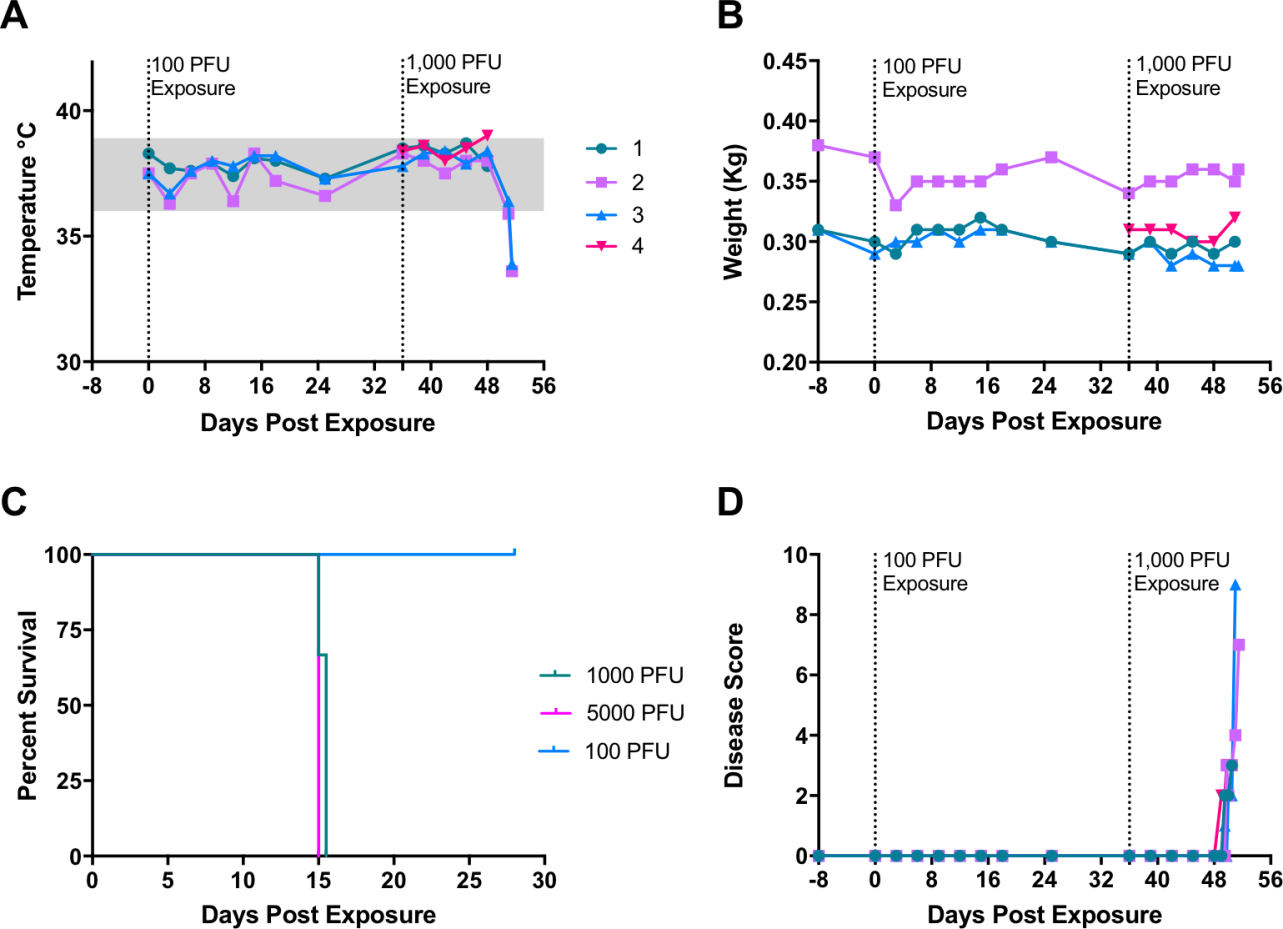
Clinical assessment of marmosets exposed to monkeypox virus. Animal 1–3 were exposed once on Day 0 with an intranasal dose of 100 PFU and subsequently re-exposed with 1000 PFU on day 36. Rectal temperature, (A), and body weight (B), were evaluated every 3 days under sedation. Survival outcome, (C). Animals were evaluated for recumbence, unresponsiveness, and dyspnea and given a disease score, (D). Three males (#1, #2, and #4) and one female (#3) were used in this experiment.

**Fig 2:**
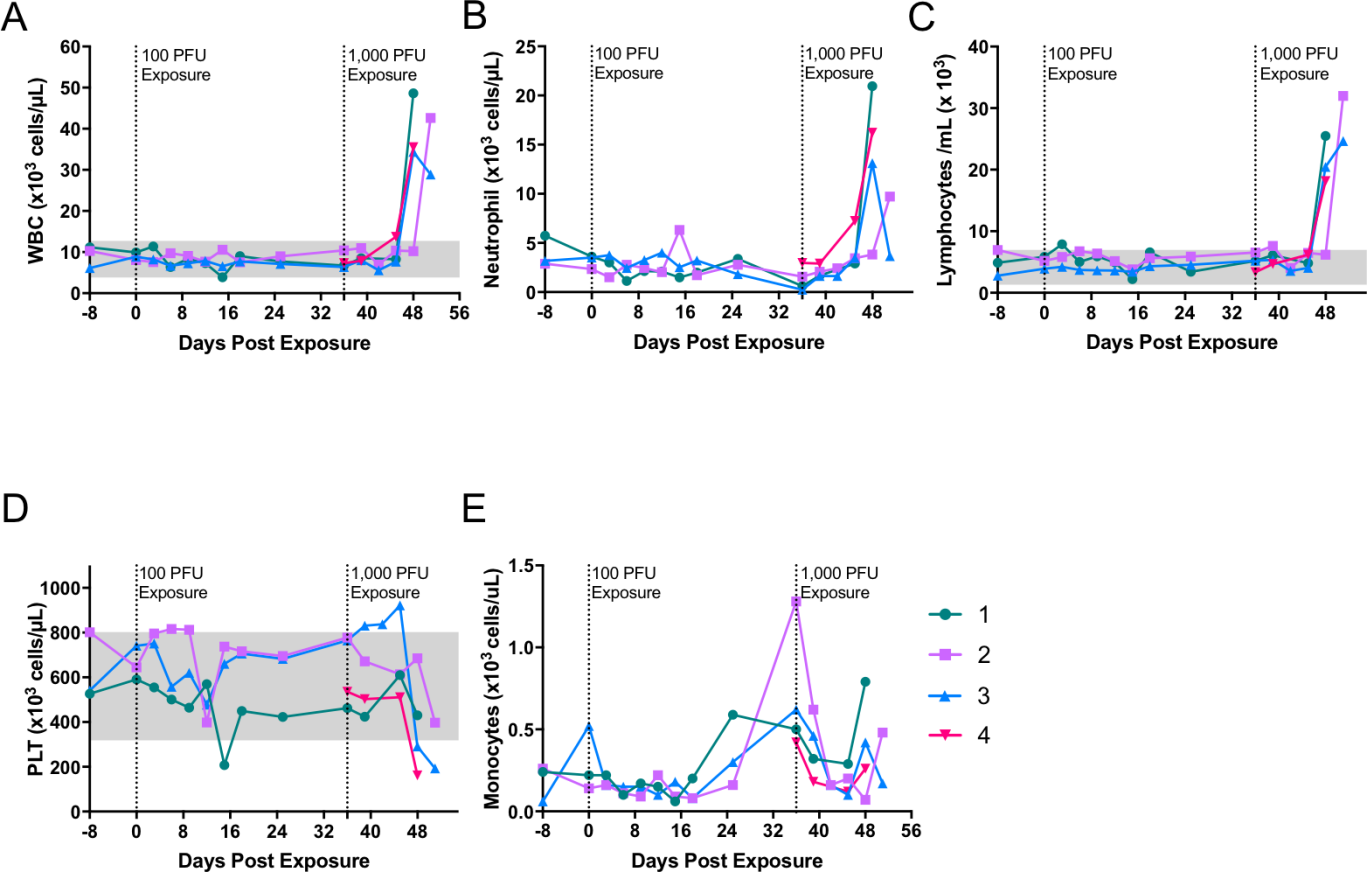
Hematology of marmosets intranasally exposed to monkeypox virus. Marmosets were exposed to 100 pfu (days 0) and later exposed (day 36) with 1000 pfu (animal #s 1, 2, and 3). Concomitantly, a naïve animal (#4) was exposed to 5,000 pfu of monkeypox virus. White blood cells (WBC) (A), neutrophils (B) and lymphocytes (C) all increased above baseline values, whereas platelets (PLT)(D) tended to drop. Monocytes (E) tended to initially drop after exposure, but rebounded or increased between days 9–15 post exposure. Data suggest that animals were not infected with 100 pfu, but were at the two higher doses. Shading represents in-house normal values for each parameter (Mucker, et al. 2015). Data was acquired using a Hemavet HV950.

**Fig 3:**
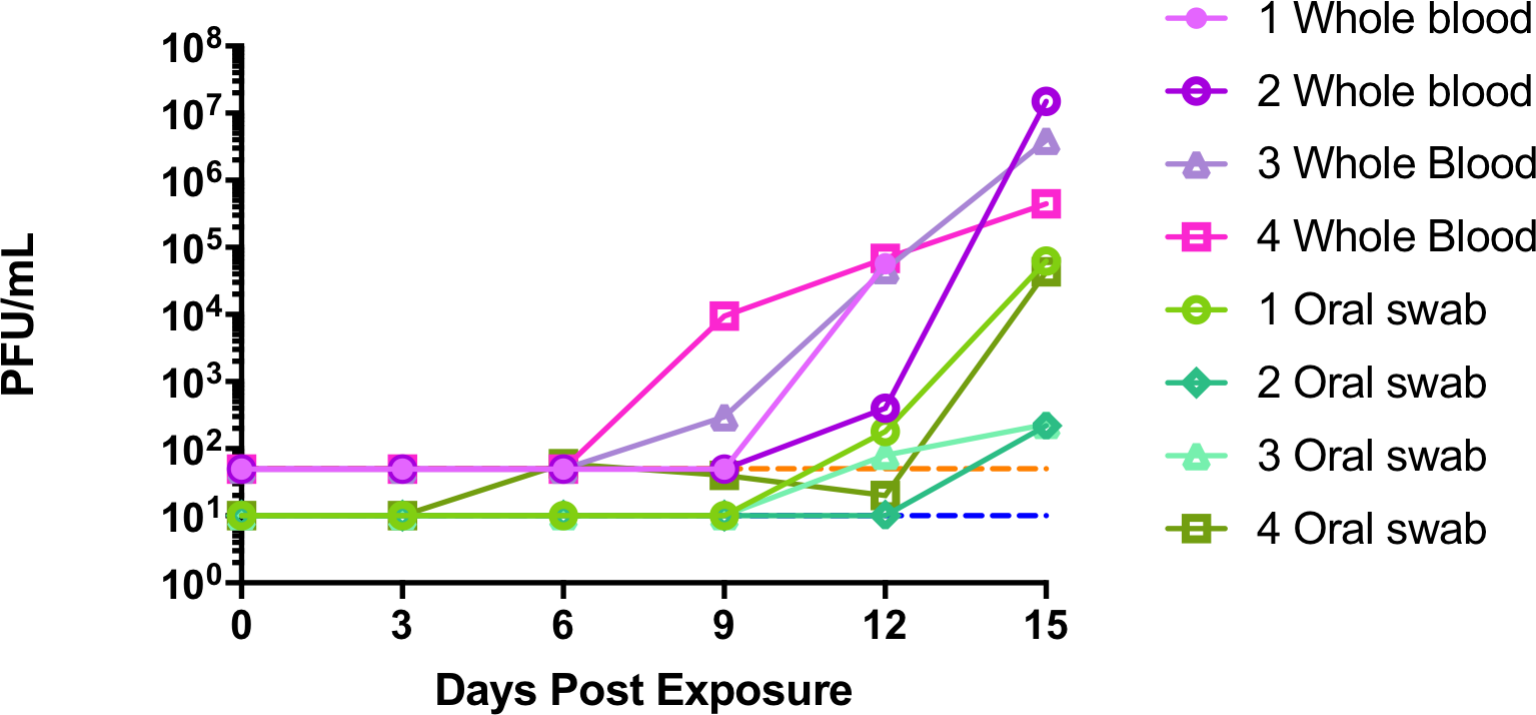
Viremia and viral shedding in marmosets exposed to intranasal monkeypox virus. Whole blood and oral swabs were processed and titrated onto BSC-1 cells. Two of four animals had detectable levels of virus in blood samples on the Day 9. Virus from oral swabs was detectable as early as day 6 (high dosed animal #4). Limits of detection for whole blood (dashed orange line) and for oral swabs (dashed blue line). All animals were positive to varying extents. Notice the onset of oral shedding relative to viremia.

Our goal for these initial experiments was to determine the dose of intranasally administered monkeypox virus required to cause disease in marmosets. We previously showed that marmosets exposed to low titer monkeypox virus administered intravenously was able to initiate severe disease [21]. Based on these findings, we decided to start with a low dose intranasal challenge. In our first experiment, three marmosets were exposed to monkeypox virus at a target dose of 100 plaque forming units (PFU). There were no clinical signs of illness in these animals (Figs 1 and 2). Hematology was normal with the exception of a reduction in platelets for animal #1 on Day 15. This was most likely an aberration (e.g., clot), as there were no other signs of illness and platelet values were within normal range on subsequent sampling days (Fig 2). Blood and oral swabs virus titrations were also negative before proceeding to our next iteration (Fig 3).

Because there were no indications of disease, we chose to re-expose the three marmosets at a higher dose (1000 PFU). A fourth marmoset, #4, was included and exposed at an even higher dose (5000 PFU). All animals displayed signs of illness and were either euthanized or succumbed to disease 15 days post exposure (Fig 1). In general, marmosets were clinically normal through day 12. Body temperatures tended to remain constant until the last two days (Days 14 and 15), at which point they dropped (Fig 1A). The single animal that was exposed at a dose of 5000 PFU, #4, had a temperature increase above the normal range. This increase was about 0.5°C above the pre-exposure temperature. Body temperatures began to decrease in animals that survived to Day 15 (animal #2 and #3). Body weights did not fluctuate much throughout the post-exposure phase (Fig 1B).

Slight changes in behavior were observed on Day 12, but animals did not meet our criteria for disease scoring until Day 13. The higher dosed animal #4 was the first to score using the criteria (Day 13, a.m.), based on observed unresponsiveness and dyspnea (Fig 1C). The three other marmosets scored throughout Day 13. Two of the four marmosets (#3 and #4) experienced varying degrees of dyspnea, with animal #3 exhibiting greater difficulty breathing. The disease progressed in all animals, with the marmosets becoming increasingly unresponsive and recumbent until they were either euthanized or succumbed to disease (Fig 1C and 1D). Other signs included, in order of frequency: Sensitivity to light, edema, and nasal discharge. All animals had edema around the eyes and some signs of photophobia. A single animal, #4, had significant nasal discharge.

**Fig 4:**
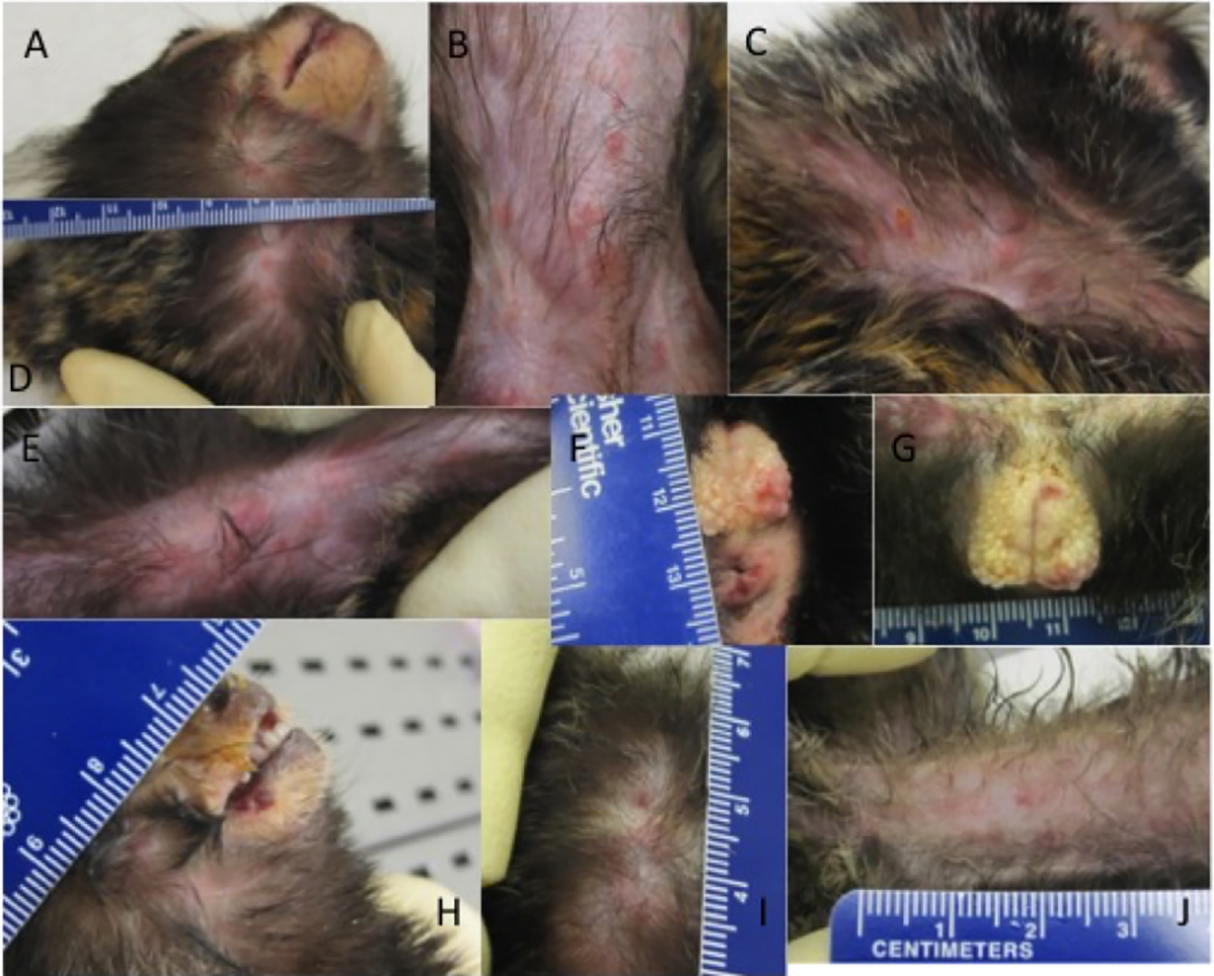
Rash in marmosets exposed to intranasal monkeypox virus. A rash was observed in two of the four marmosets, animal 3 (A-G) and animal 4 (H-J). Shown are the head (A), abdomen/groin (B), side of chest and left arm (C), left armpit with enlarged lymph node (D), and urogenital region (F and G) of animal 3. From animal 4, mouth and copious nasal discharge (H), back of skull (I), and lower abdomen (J). Notice that the lesion on animal 4 are in a papular/vesicular stage of rash with few pustules whereas animal 3 has a macular/papular rash that had flattened has areas hemorrhage (petechial in nature).

Skin lesions were present on two of the four animals (Fig 4). On animal #4, two focal papules were observed on Day 12. By Day15 there were a total of nine lesions, seven of which had a hemorrhagic (petechial) appearance (Fig 4A and 4C) and two were scabbed (Fig 4B). Animal #3 had a total of 57 lesions observed on Day 15. In contrast to animal #4, animal #3 had a vesicular/pustular rash covering most of the body (Fig 4D, 4E and 4F). At the time of death, there were no skin lesions on animals #1 and #2.

Analysis of whole blood samples revealed increases in white blood cells and decreased platelet counts (Fig 2). Both lymphocytes numbers and neutrophils were greatly elevated in 3 of 4 animals on Day 12 and all animals by Day 15 (Fig 2B and 2C). Platelets counts were markedly decreased in 3 of the 4 animals (#’s 2, 3 and, 4). Decreases in platelets were observed in animals #3 and #4 on Day 12 (Fig 2D). In total, platelets were reduced from baseline by approximately 45–70% in animals #2, #3 and #4. Monocytes tended to decrease after exposure, but subsequently rebounded between days 9 and 15 (Fig 2E).

Virermia and oral shedding were assessed by plaque titration of whole blood and oral swabs. Using whole blood samples, all animals were viremic by Day 15 (Fig 3). Animals #3 and #4 had detectable viable virus on Day 9 and animal #1 and #2 on Day 12. Animal #2 had observed viremia only on Day 15, and had the highest levels of circulating virus. Analogous to whole blood, all animals had infectious virus in the oral cavity by Day 15. The earliest detectable shedding was animal #4, which had detectable virus on Day 6. Of the remaining animals, #1 and #3 had detectable levels on Day12 and #2 on Day 15. Animal #1 and #4 had similar higher titers on Day 15, having approximately 2 logs more virus than #2 and #3 (Fig 3).

### Prophylactic treatment with a monoclonal antibody cocktail

After determining a dose of virus that causes severe disease from the previous experiments, we next wanted to explore whether or not we could protect the marmosets from such a disease. A group of three marmosets (#8, #9, and #10) were treated subcutaneously with a 1:1 mixture of monoclonal antibodies c7D11 and c8A at 20 mg/kg (per antibody) 24 hours previous to monkeypox virus exposure. As controls, one marmoset was treated with an equivalent dose of isotype matched (non-orthopox virus targeted, BioFactura) monoclonal antibody (#5) and two marmosets were treated with an equivalent volume of PBS (#6 and #7).

**Fig 5:**
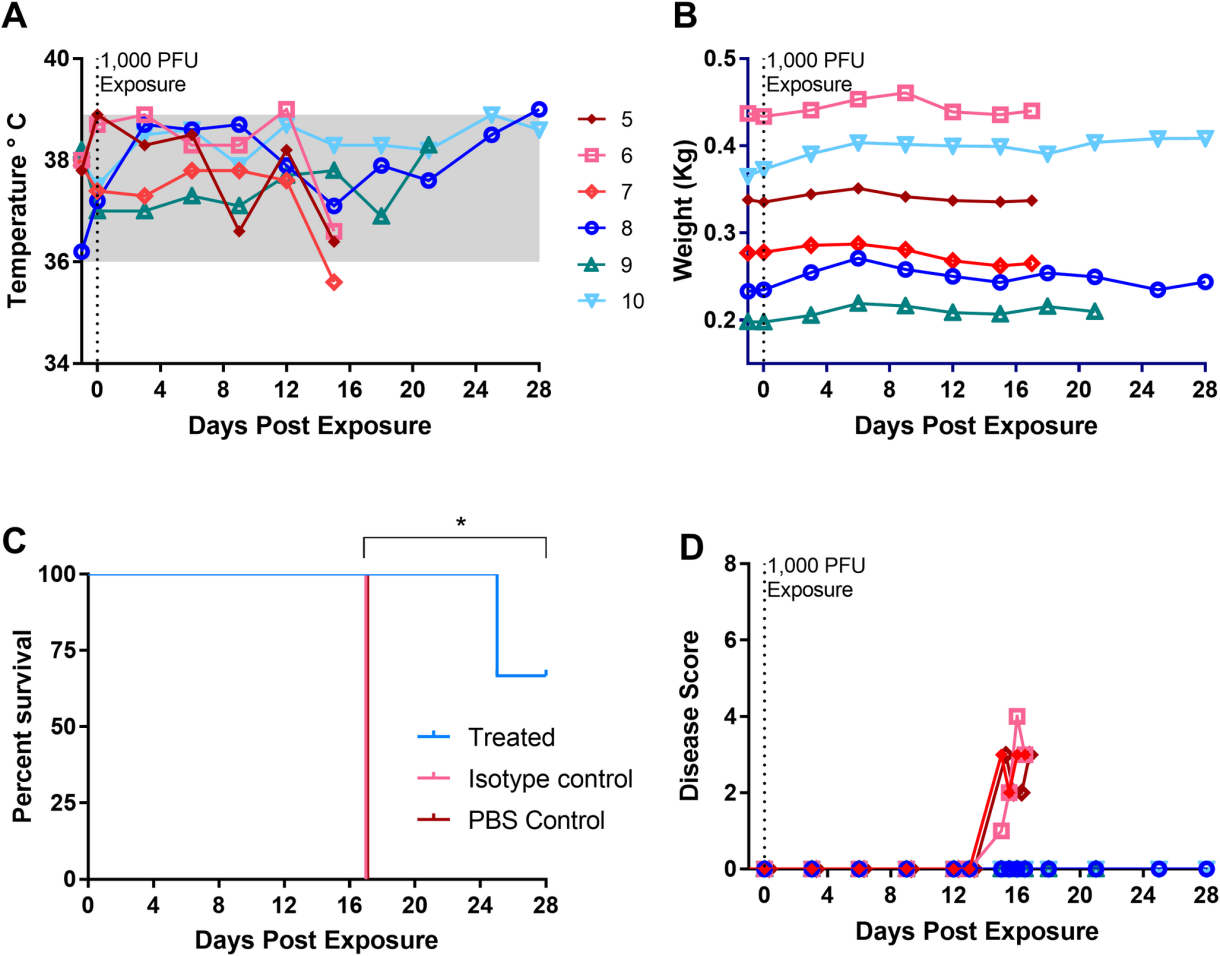
Clinical assessment of antibody treated marmosets exposed to monkeypox virus. Marmosets were sedated every third day at which time body temperature (A), weights (B). Disease score (D) was captured daily. All control animals succumbed to disease, while animals that received targeted antibody treatment survived until at least Day 25 (C). Asterick denotes significance in survival curves between untreated and mock treated versus treated (Log rank, p = 0.0253). Three females (#’s 5, 6 and 8) and three males (#’s 7,8, and 9) were used in this study.

**Fig 6:**
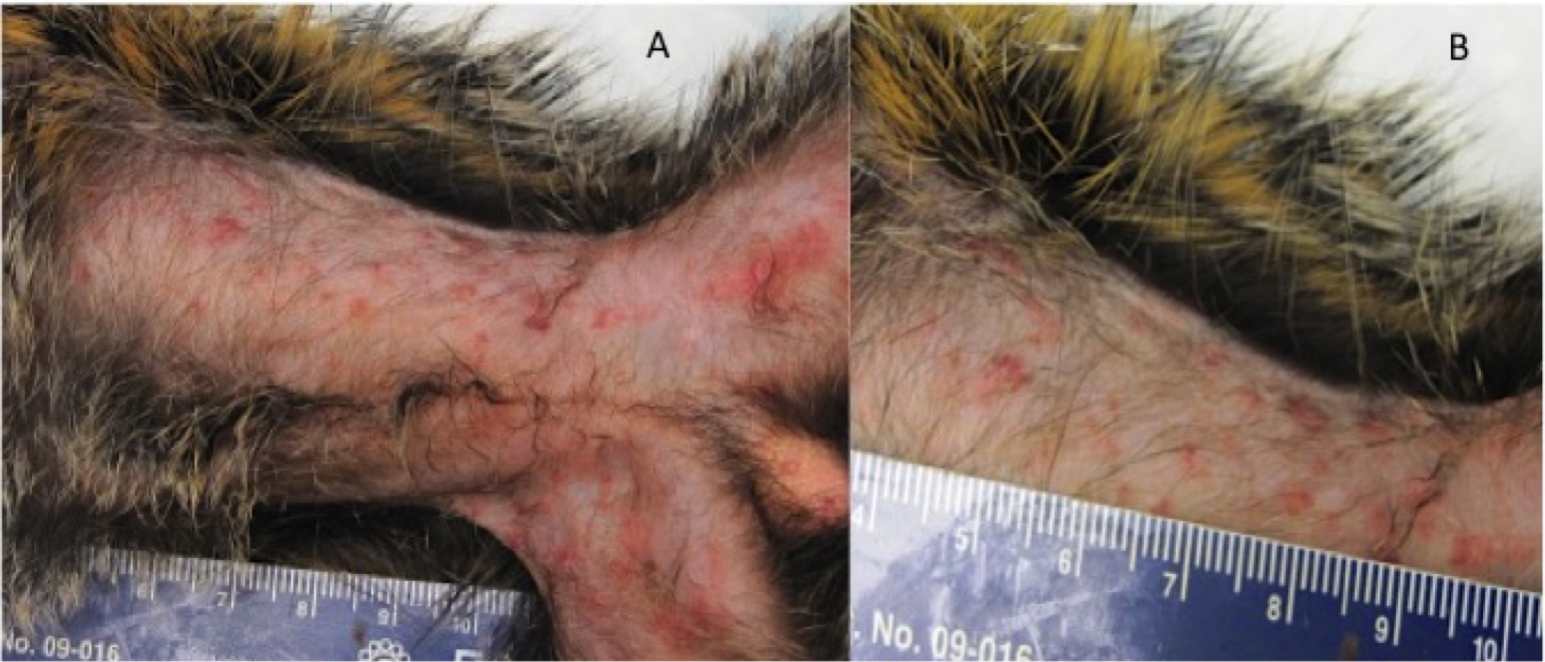
Skin of a mock treated marmoset exhibiting a classical smallpox rash. Previous to exposure with 1000 PFU of monkeypox virus, animal 5 was treated with non-pox-virus specific antibody. This animal manifested a typical smallpox rash as can be seen on the exposed (hairless) portions of the abdomen and groin (A) a zoomed in portion of the abdomen is shown in B.

The disease course in the control animals was similar to our initial experiments. There were no significant increases in temperature, but animal’s had slight decreases on Day 15 (Fig 5A). Body weights were constant throughout the experiment with only minor fluctuations (Fig 5B). Animals exhibited behavioral changes on Day 13, but did not score (disease scoring criteria) until three days before succumbing to the disease (Fig 5C). A macular/papular rash was apparent on animal #6 and #5 on Day 15 with approximately 20 and 139 lesions counted, respectively. The rash of animal #6 became hemorrhagic in nature whereas the rash of animal #5 progressed in both morphology and magnitude. Animal #5 had and estimated lesion burden of 299 lesions (Fig 6). All control animals succumbed to disease on day 17 post exposure (Fig 5D).

Animals prophylactically treated with c7d11 human-mouse chimera and c8a human-chimpanzee chimera antibodies did not exhibit signs of any illness throughout the critical period, that is, when the untreated animals were showing signs of disease or died (Days 13–18). With the exception of animal #9, all treated animals survived the exposure (Fig 5D). Animal #9 did not exhibit any signs of disease until day 24, when it was observed that the animal had eaten very little. The next morning the animal was found dead with blood around the animal’s rectum and in the stool. There was also a single lesion on the chin of the animal. There is statistical significance between treated (n = 3) and un/mock-treated (n = 3) surivival curves (p = 0.0253). ().

We further explored the implications of the circulating and shed virus by assessing the virus susceptibility to monoclonal antibodies. To do this, we performed a plaque reduction assay (varying virus titer, constant antibody concentration) with c7D11and virus collected using the oral swab from animal #9 on Day 25. We found that 10μg/mL of c7D11 greatly reduced the virus as follows: >1250 PFU/mL reduced by greater than 89%; 210 PFU/mL was reduced by 96% and 55 PFU/mL was reduced by 100%. These experiments allowed us to conclude that the virus was indeed monkeypox virus and also that the shed virus was itself not resistant to our most potent therapeutic component (c7D11). With limited plasma sample, we also looked at levels of c7D11 and c8A on Days 0, 12, and 21 by ELISA. Animal #9 had approximately 1/3 less c7D11 in the plasma as the other two animals and, 15% less c8A. Differences between animal #8 and #10 were slight in levels of c7D11 and c8A (376 vs. 364 μg/mL and 195 μg/mL vs 196 μg/mL, respectively). Differences became apparent (or more apparent) on Days 12 and 21. For instance, animals #8 and #9 had c7D11 levels amounting to 36% and 8% on Day 12 as compared with animal #10, and 19% and 2.3% on Day 21. For c8A, this equated to 85% and 33% for Day 12 and 5%, and 41% for Day 21 (for related data, see results section concerning the magnitude and longevity of the antibody below).

**Fig 7:**
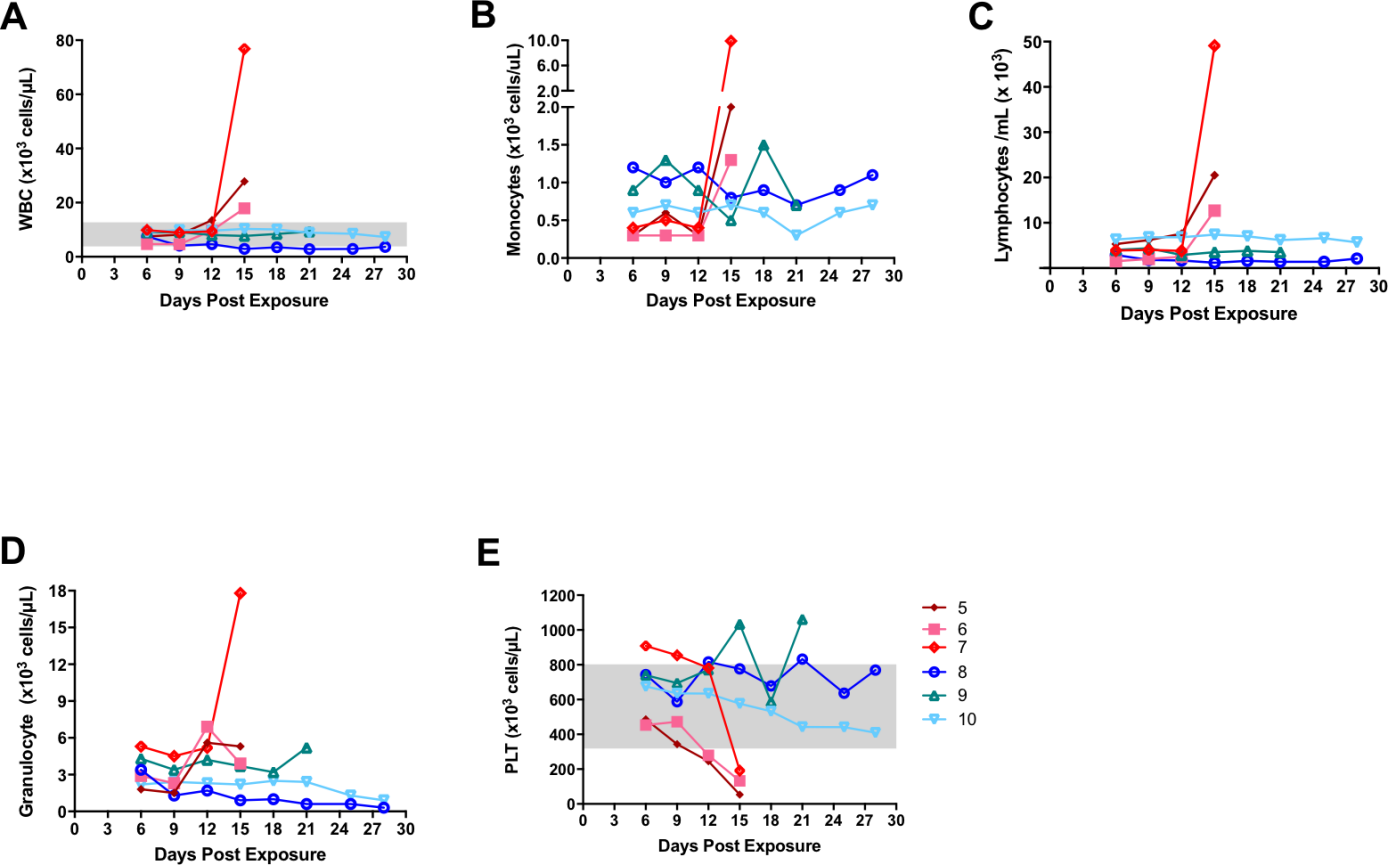
Select hematology from marmosets, some treated, then exposed to intranasally to low dose monkeypox virus. Prior to challenge, animal 5 was treated with an isotype control antibody, while animals 6 and 7 were treated with PBS. Animals 8, 9, and10 were treated with monkeypox targeted antibody. White blood cells (WBC) (A), lymphocytes (B), monocytes (C) and granulocytes (D) were elevated, and platelets (PLT) (E) decreased in most of the control animals whereas treated animals did not vary much from earlier time points. Data was collected using a Coulter AcT Diff3.

Hematology in control animals was similar to our previous experiment. White blood cells and lymphocytes numbers were increased on the final bleed day (Day 15) before succumbing to the disease (Fig 7A and 7B). Unlike the last experiment, monocytes were increased (Fig 7C). Granulocytes increased in two of the three animals, #6 and #7 on days 12 and 15, respectively. Little fluctuation was observed in the treated animals. Monocyte numbers seemed to decrease and subsequently increase for animal #9 on days 15 and 18. When compared with earlier time points the decrease and subsequent increase is approximately 0.4 and 0.6, respectively. Similar to our first experiments, mock treated animals had a reduction in platelets 2–3 days previous to death (Fig 7E). Treated marmosets platelet values were within normal range. Animal #9 did have fluctuations above the normal range. Given the variation in the data for this animal, it is difficult to assess whether this flucation was related to infection.

**Fig 8:**
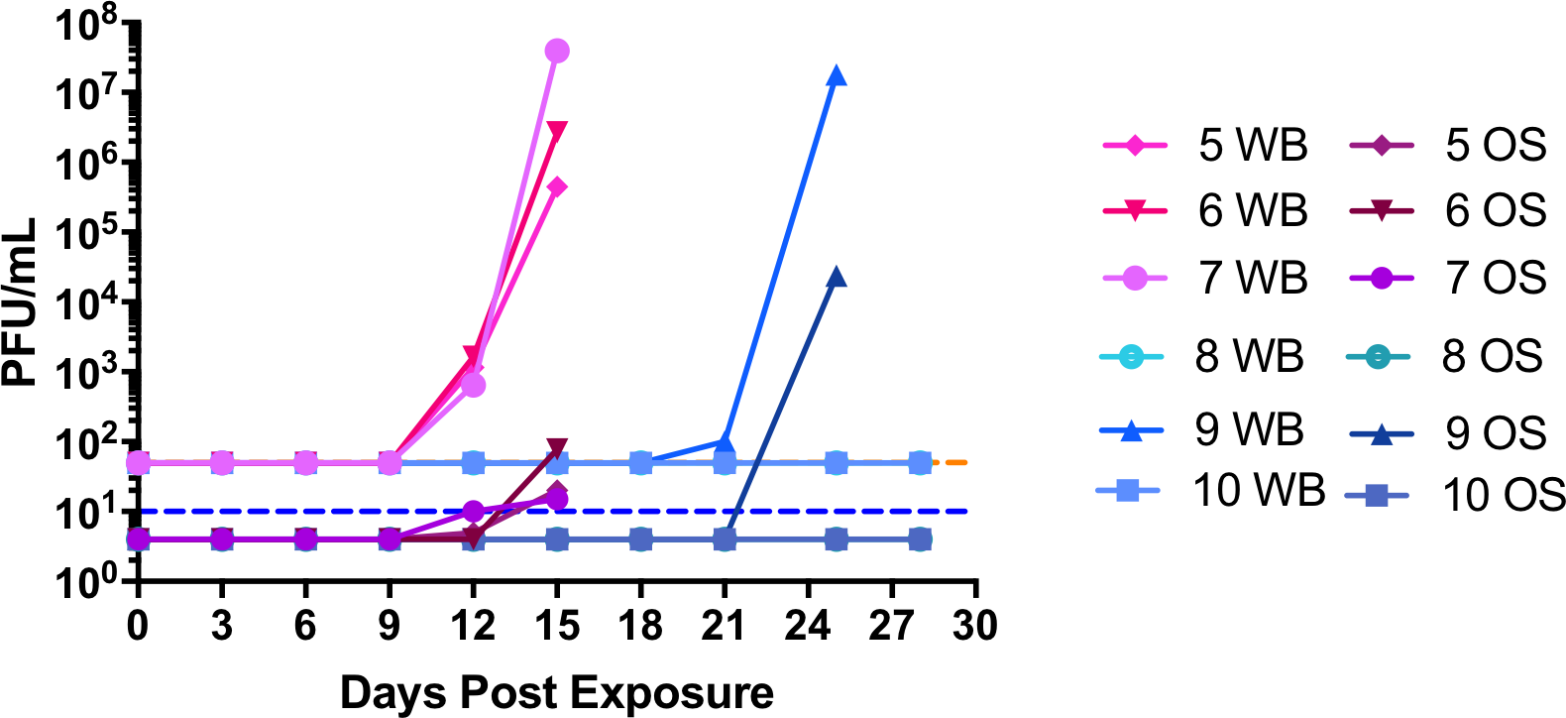
Prophylactic treatment with c8A and c7D11 prevents or delays virermia and viral shedding. EDTA whole blood (WB) and media from oral swabs (OS) were inoculated on to BSC-1 cells, stained, and plaques enumerated five days later. Treated animals (shades of blue); Mock or off-target treated (shades of pink). Limits of detection for whole blood (dashed orange line) and for oral swabs (dashed blue line).

All control animals were viremic at Day 12 post exposure (Fig 8). Animal #7 had over 7logs of infectious virus on Day 15, the most virus detected in all the control animals. Of the treated animals, animal #9 was the only animal to have detectable virus. Low levels of virus (10 PFU) were detected on Day 21. Post mortem blood that had pooled in the heart (Day 25) had levels comparable to control animal #7. The other two treated animals (#8 and #10) were below the limit of detection of our assay for all blood samples examined.

Infectious virus was detected in oral swabs on Day 12 (animals# 5 and #7) and Day 15 (animal #6). Low levels of virus were detected (less than 100 PFU) at these time points and, interestingly, there was not an appreciable increase between Day 12 and Day 15 for the animals that were positive on Day 12 (Fig 8).

### Magnitude and longevity of a cocktail containing c7D11 and c8A monoclonal antibodies in marmosets

**Fig 9:**
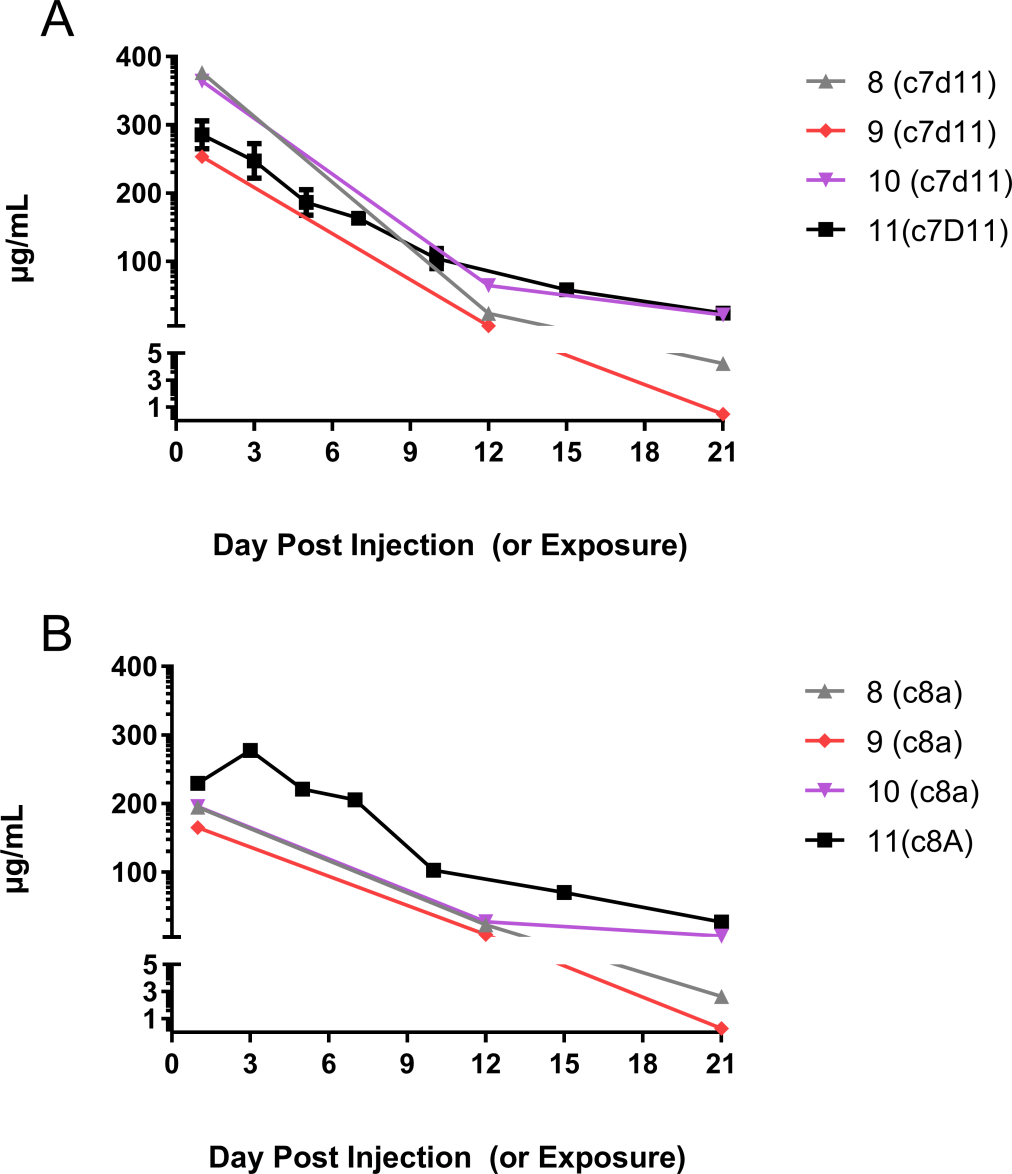
Levels of c8A and c7D11monoclonal antibodies in infected and uninfected marmosets. Marmosets were subcutaneously injected with a 1:1 mixture of a cocktail containing c8A and c7D1 (20mg/Kg of each antibody per animal). A single animal was not exposed to monkeypox virus (#11). Of the exposed animals, Days 1, 12 and 21 were analyzed for the presence of c7D11 (A) and c8A (B). For infected animals #8, #9 and #10, days are relative to the day of exposure. For animal #11, days are relative to subcutaneous injection of the antibody.

To assess the relative levels of monoclonal antibodies c7D11 and c8A at the time of exposure and persistence over time in uninfected animals, we recapitulated the efficacy experiment by injecting 20mg/Kg (of each antibody, as a cocktail) into a single marmoset (Fig 9). Representing the peak amounts captured by sampling, there detected 285 ug/mL of c7D11 at twenty-four hours and 277 μg/mL of c8A at 48 hours after subcutaneous injection. Both antibodies were detectable until the last day of collection (86 days) with approximately 23 ng/mL of c7D11 and 3ng/mL of c8A. The half-life for both antibodies fell between 7 to 10 days.

## Discussion

Here we present for the first time the successful treatment of a monkeypox virus exposed marmoset. The antibodies tested are known to target both morphogenic forms of the virus: the MV, in the case of c7D11, and the EV, in the case of c8A. Previously, 7D11 was studied as a full mouse monoclonal antibody [13, 23] and c8A had a slightly different human heavy chain constant region[14]. In our studies, we evaluated novel human-chimeric forms of the antibodies. We found that disease was prevented in 2 of 3 marmosets, and there was a delay (approximately 8 days) to signs, virological indications, and death in the third marmoset. Based onour data, the third animal most likely succumbed because of lower initial levels of antibody. The largest differences were observed with our most potent antibody, c7D11. Interestingly, there were differences observed between all three treated animals at days 12 and 21post exposure. Whether the antibody is being turned over by the host or consumed by a viral infection is unknown. Comparing these data to our data from an uninfected animal suggests varying degrees of infection based on decreases in monoclonal antibody levels. Since only one uninfected animal was utilized to evaluate monoclonal antibody levels, this does not rule out different depletion rates of antibody treatment from the animals. In either case, the captured data will help center in on an efficacious dose based on plasma/serum levels.

Using a small number of marmosets, we found that a relevant dose of 1000 PFU was sufficient to cause uniformly severe disease. Exposure with 100 PFU was not sufficient to cause disease. Other than a single hematological analyte (platelets) from a single animal, all parameters measured were normal. This includes a lack of detectable virus in the samples. From our data, the most likely scenario is that monkeypox virus failed to infect and instead was mechanically (and not immunologically) cleared. The evidence for this is that there have been no reported experimental survivors that have exhibited clinical disease or presented with hematological/physiological manifestations (i.e., no evidence of subclinical infections) [21]. Also, there is data where lower doses cause severe disease in marmosets. Doses as low as 48 PFU administered intravenously were capable of causing infection and death [21], adding credence to the theory that the lack of disease using 100 PFU was related to administration and, most likely, not susceptibility. Although we did not optimize the intranasal delivery (e.g, volumes, head tilt), this could possibly lower the concentration of virus required to elicit disease.

During the incubation (pre-prodromal period) for naturally acquired smallpox, it is thought that a primary viremia occurs from the local lymph tissue which ultimately seeds the hematopoietic system [24]. A secondary viremia follows concomitant with the febrile period approximately 12.5 days after infection [25]. Screening of throat secretions/washes for infectious virus suggested smallpox afflicted individuals are contagious during and beyond the febrile period [26–28], With less frequency, infectious virus was detected previous to fever [28]. Shortly after the onset of fever, a centrifugal, progressive cutaneous rash develops. Although this sequence of events is prototypical of smallpox, the clinical course of smallpox was quite variable (e.g., hemorrhagic, modified) [24].

**Fig 10:**
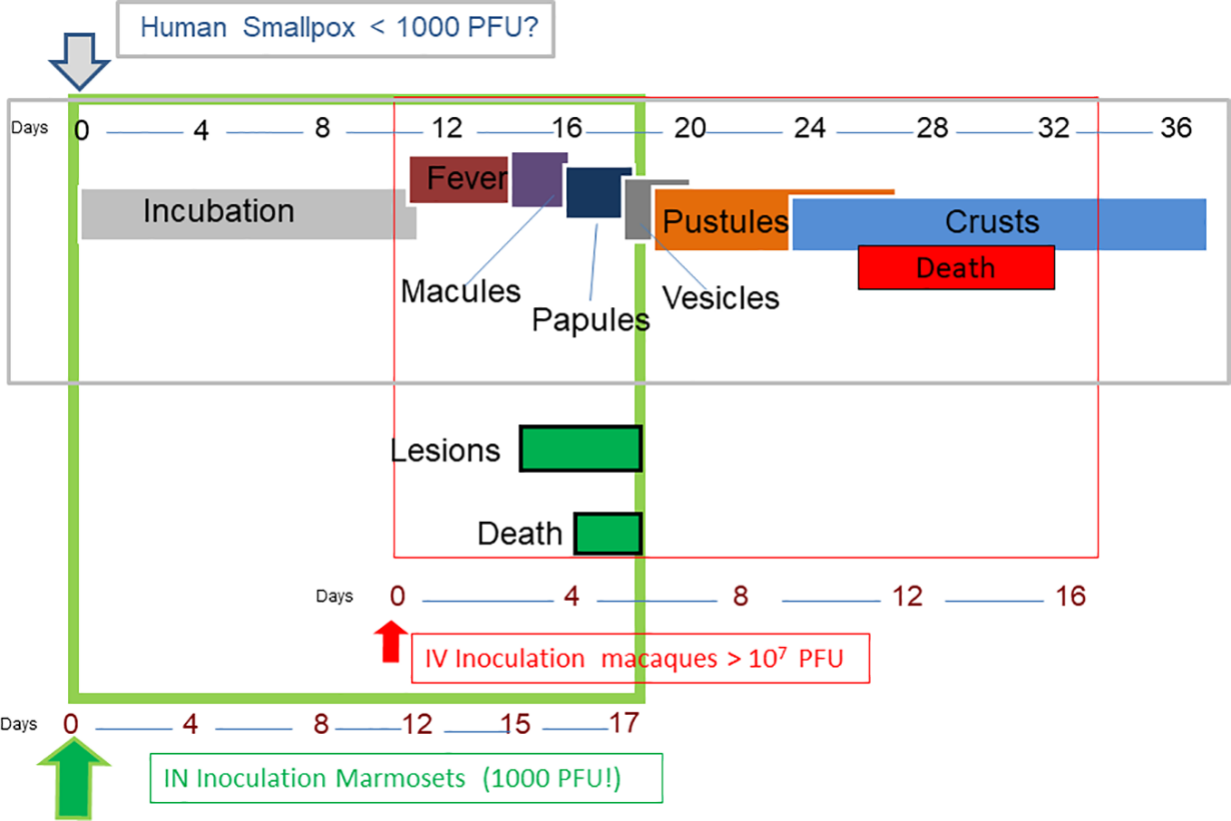
Comparison of the clinical course of smallpox and monkeypox in humans and infected nonhuman primates. To elucidate both the resemblance to human smallpox disease and how our model relates to the “gold standard” of NHP models for poxvirus countermeasures, the figure depicts the human disease (grey outlined box), the IV monkeypox-macaque model (red outlined box) and the IN monkeypox-marmoset model (outlined in green). Arrows are color coded by host and indicate the point of exposure. Days (in the form of a number line) relative to exposure are given for each Human disease manifestations are temporally identical to IN exposed marmosets to include viremia and oral shedding (not shown-see discussion), but fever has not yet been detected in marmosets (IV = intravenous; IN = intranasal). This Figure is a modification of Figure 34 from Mucker, 2014 [36].

Of the six mock or un-treated marmosets exposed to 1000 PFU of monkeypox virus, the sequence and temporal onset of disease events were analogous to those delineated above for human disease (Fig 10). For instance, we detected a viremia that either preceded or was concomitant with infectious oral swab samples at an average onset of 11 days and 13 days, respectively. These data suggests that virus in the oral cavity is most likely a consequence of viremia (either an undetected primary or secondary) seeding these tissues, as opposed to virus propagating at the site of initial exposure. Viremia also proceeded rash (onset average of Day15) suggesting a similar seeding event. Physical manifestations of disease were observed at an average of 13.4 days. One of the six animals presented with a slightly elevated temperature (on Day 12). Although the temperature data fits appropriately in the model of human smallpox (Fig 10), more and better data is required before making a stronger assertion. More convincing data could be gathered through the use of telemetry.

Since animal #4 was exposed to 5000 PFU, we will have to consider it separately from 1000 PFU, and, given it is the only animal exposed at the dose, not infer too much from the data. The onset of viremia and detection of infectious virus on oral samples were earlier than the 1000 PFU animals. Sequentially opposed to the 1000 PFU animals, oral swabs were positive for infectious virus on Day 6 and viremia was not detected until Day 9. This could be potentially be explained by the sensitivity of the titration assays or that oral tissues are seeded prior to, not by, the viremia we detected in the blood. There were no real differences in magnitude in any of the parameters measured (e.g., titers, temperature, lesions).

Unlike the monkeypox virus intravenous marmoset model, a visible cutaneous rash was not a consistent feature of the disease in the intranasal marmoset model [21]. In fact, three control animals of seven did not have a rash, but rash was more characteristic of ordinary smallpox and the lesion burden was far greater than reported by Mucker, et al 2015 [21]. Of the four, two had a progressive macular/popular rash with a single animal (#5) that had a near confluent rash at the time of death. In comparison, the intravenous marmoset model had a rash that was mainly hemorrhagic and resembled early type hemorrhagic smallpox. The variability between individuals in terms of presentation of skin lesions, and correlating outcomes, has been well described for smallpox. For example, the rash phenotypes seen in our study, hemorrhagic and confluent, have been associated with more severe disease and higher mortality. The reason for these differences in humans could be dose or strain related, but it is generally agreed that host genetic predisposition is most likely the most important factor. Doses administered to each marmoset (per experiment) were prepared from the same pool of virus, reducing the chance of a difference in the concentration of the stock being implicated for variability in disease (e.g., rash).

Whether adaptive and/or innate response(s) is insufficient in marmosets and leads to more severe disease is unknown and will require more experimentation. Severe disease in mice infected with ectromelia virus has been linked to Natural Killer cells [29] and low numbers of NK cells and T cells in CAST/EIJ mice infected with monkeypox virus [30, 31] For smallpox vaccinations, containment of the spread of lesions are thought to be cell mediated [32, 33]. Future studies of monkeypox in marmosets could expand on these ideas.

Interestingly, there appears to be a gender effect related to cutaneous lesion burden (Table 1). Females had more lesions than males. This was also accompanied by lower maximum observed viremia and lower oral shedding, on average (Table 1). This suggests some immune control or dampened replication of the virus in females that is sufficient enough, at least temporally, to allow for the formation of lesion. In terms of viremia, we also see this phenomena in the only male that developed lesions. More severe forms of smallpox, especially early-type hemorrhagic, are associated with much higher levels of infectious virus in the blood and oral washings and a low, or nonexistent, humoral response (reviewed by [24]). As mentioned, hemorrhagic forms of smallpox disease do not form a typical rash (or do so short term) most likely due to an overwhelming infection killing the host before forming lesions. Three of four males in our studies died before lesions developed. Additionally, the only male to develop lesions had a hemorrhagic presentation.

**Table 1:** Gender comparison of total skin lesion counts, viremia titers, and oral titers.

	Lesions (total per animal)	Average Viremia Maximum Observed (PFU/mL)*	Average Oral Maximum Observed (PFU/mL)*	N
Male	0,0,0	1.81E+07	20912	3
**Male**	**9**	**4.45E+05**	**43000**	**1**
Female	299+, 57, 20	2.37E+06	109	3

Bold = animal exposed to 5000 PFU

* Average calculated from Day 15 values

How this applies to the outcome of smallpox and human monkeypox is difficult to conclude. It is well known that in humans, pregnant females were significantly more likely to develop more severe form of the disease (hemorrhagic disease), but in the absence of pregnancy there was no clear gender effect on severity or outcome. Others have reported differences in immune response after smallpox vaccination. For instance, it has been reported that in humans, females generate a higher neutralizing response to Dryvax [34]. The opposite effect has been reported for IMVAMUNE but the IMVAMUNE vaccine is administered differently (subcutaneous administration, as opposed to scarification with Dryvax) and is replication incompetent in mammalian cells, making a direct comparison impossible. [35]. Given the scope of the study and the limited volume of sample, such immunological studies were not performed. Therefore, future studies should look at whether these differences are real and, if they are, help define a mechanism.

Both human monkeypox and smallpox have similar disease manifestations and were difficult to decipher in the age of smallpox. In the eradication era, this was not an issue from the clinical perspective as the vaccine provided protection from both monkeypox and smallpox. Now that the global population is becoming more immunologically susceptible to monkeypox, there are more monkeypox outbreaks with more associated cases of monkeypox. Therefore, a global predicament is more likely than before and an adequate level of preparedness should be sought. Concerns for monkeypox should not completely overshadow the threat of accidental or purposeful reintroduction of smallpox. Because of the relatedness of the monkeypox and variola viruses, therapeutic and vaccine strategies can be bundled for both indications. With the advent of the intranasal monkeypox virus model in marmosets, the entire disease course of monkeypox and smallpox can be accounted for by nonhuman primate models utilizing a virus known to cause severe disease in humans (Fig 10). For smallpox medical countermeasures, the monkeypox virus models are in lieu of a test system that incorporates variola virus. Variola virus has never been tested in marmosets, but, given the results of our studies, this would be the next logical step.
